# Astrocyte Heterogeneity: Impact to Brain Aging and Disease

**DOI:** 10.3389/fnagi.2019.00059

**Published:** 2019-03-19

**Authors:** Isadora Matias, Juliana Morgado, Flávia Carvalho Alcantara Gomes

**Affiliations:** Laboratory of Cellular Neurobiology, Institute of Biomedical Sciences, Federal University of Rio de Janeiro, Rio de Janeiro, Brazil

**Keywords:** astrocyte, glial reactivity, aging, heterogeneity, neurodegenerative diseases

## Abstract

Astrocytes, one of the largest glial cell population in the central nervous system (CNS), play a key function in several events of brain development and function, such as synapse formation and function, control of neurotransmitters release and uptake, production of trophic factors and control of neuronal survival. Initially described as a homogenous population, several evidences have pointed that astrocytes are highly heterogeneous, both morphologically and functionally, within the same region, and across different brain regions. Recent findings suggest that the heterogeneity in the expression profile of proteins involved in astrocyte function may predict the selective vulnerability of brain regions to specific diseases, as well as to the age-related cognitive decline. However, the molecular mechanisms underlying these changes, either in aging as well as in brain disease are scarce. Neuroinflammation, a hallmark of several neurodegenerative diseases and aging, is reported to have a dubious impact on glial activation, as these cells release pro- and anti-inflammatory cytokines and chemokines, anti-oxidants, free radicals, and neurotrophic factors. Despite the emerging evidences supporting that reactive astrocytes have a duality in their phenotype, neurotoxic or neuroprotective properties, depending on the age and stimuli, the underlying mechanisms of their activation, cellular interplays and the impact of regional astrocyte heterogeneity are still a matter of discussion. In this review article, we will summarize recent findings on astrocyte heterogeneity and phenotypes, as well as their likely impact for the brain function during aging and neural diseases. We will focus on the molecules and mechanisms triggered by astrocyte to control synapse formation in different brain regions. Finally, we will discuss new evidences on how the modulation of astrocyte phenotype and function could impact the synaptic deficits and glial dysfunction present in aging and pathological states.

## Astrocytes: An Overview

The term “astrocytes” comprises a heterogeneous group of non-neuronal cells, involved in fundamental functions of the central nervous system (CNS). Even though astrocytes tile the entire brain, these cells have not always been considered active partners of neurons in the transfer of neural information as they are now, in the beginning of the XXI century.

In the XIX century, Virchow coined the term *nervenkitt* (neuroglia), to refer to the passive, connective elements in the brain, in which the other elements, the excitable ones, were embedded (Somjen, 1988). Further discoveries about the nature of neural cells came in the end of the XIX century, with new techniques of tissue staining developed by Italian physician and cytologist, Camillo Golgi and the Spanish neurohistologist, Ramón y Cajal. Golgi and Cajal were the first to highlight that neuroglia and nerve cells represented different populations and to further identify a variety of glial shapes and forms, as well as the glial network formed by these cells and other non-neuronal cells, such as the glial endfeet in close proximity to blood vessels (De Carlos and Borrell, 2007). At the end of the XIX century, the Hungarian anatomist and histologist, Lenhossék, introduced the term astrocyte to refer to a star-shaped glial cell; he raised the concept that even though astrocytes were electrically silent, they had functions as important as nerve cells (Somjen, 1988; Verkhratsky and Butt, 2013; Verkhratsky and Nedergaard, 2018).

In the last decades, an increasing amount of data has provided new insights on the plethora of functions performed by astrocytes. In the healthy tissue, these cells occupy unique spaces in which their extensive branching of fine processes occupy contiguous non-overlapping domains (Bushong et al., 2002). Their processes can contact synapses, other glial cells, blood vessels, and depending on the brain area, they have more specific roles. One of these key roles is in synaptic regulation, as astrocytes can act not only in the formation and maturation of synapses (Diniz et al., 2014a), but also in the maintenance, pruning and remodeling of synapses in the development, aging and diseases (Chung et al., 2013, 2015, 2016; Liddelow et al., 2017). Beyond the well-established concept of the tripartite synapse, in which perisynaptic astroglial processes are fundamental participants in the synapse, along with the pre- and post-synaptic components (Araque et al., 1999), it is now argued that the astroglial synaptic coverage could be far more extensive. It is theorized that the astroglial perisynaptic processes form a “synaptic cradle” around the synapse, embracing it, allowing the astrocyte to provide proper maintenance of the synapse, maintaining neurotransmitter, ion and volume homeostasis, releasing neuromodulators and keeping the specificity of the signaling, and providing synaptic isolation (Verkhratsky and Nedergaard, 2014, 2018). As astrocytes express a diversity of neurotransmitters’ receptors and transporters, they can control the levels and activity of several neurotransmitters such as glutamate, gamma-aminobutyric acid (GABA), adenosine triphosphate (ATP) and D-serine (Rothstein et al., 1994, 1996). Some of these molecules, also known as gliotransmitters, can be secreted by astrocytes in the synaptic space and activate neuronal receptors, allowing astrocytes to act as modulators of neuronal activity (Volterra and Meldolesi, 2005).

Emerging evidence from the last decade have strongly challenged the concept that brain function is a result of solely neuronal networks’ activity (Araque and Navarrete, 2010; Di Castro et al., 2011). Changes in synaptic function can cause the release of other gliotransmitters by astrocytes, such as thrombospondins (TSPs; Christopherson et al., 2005; Eroglu and Barres, 2010), hevin and secreted protein-acidic and rich in cysteine (SPARC; Kucukdereli et al., 2011), glypicans, and cytokines, including tumor necrosis factor-α (TNF-α; Stellwagen and Malenka, 2006) and transforming growth factor-β1 (TGF-β1; Diniz et al., 2012, 2014b). This could happen because neural activity cause excitability in the astrocyte’s membrane, triggering the release of intracellular Ca^2+^, which in turn, causes the release of some gliotransmitters (Perea and Araque, 2007; Araque et al., 2014); however, the mechanisms that govern astrocytes’ influence on synaptic activity are not completely clear yet. Further, neuronal activity has also been implicated in the generation of spontaneous syncytial signaling through calcium waves in astrocytes (Shigetomi et al., 2010; Di Castro et al., 2011), even though this is not entirely dependent on neuronal activity (Nett et al., 2002). Physiological stimulation creates a flux of Ca^2+^ ions, changing the Ca^2+^ concentration throughout cell compartments, thus generating the waves. The waves can spread out (Kanemaru et al., 2014) and propagate the signal from one cell to the other through gap junction channels (De Bock et al., 2014). This could represent a specific kind of cell-cell communication, especially for long-range information transfer in astrocytic syncytia (Scemes and Giaume, 2006). However, the proper physiological role of calcium waves and the mechanism through which it happens remains to be explored.

Besides their roles in the synaptic function, astrocytes are responsible for ion homeostasis in the CNS, regulating the extracellular levels of potassium, chlorine and calcium ions, water homeostasis and maintenance of the cellular pH. They are also responsible for providing energetic metabolites required by neurons in neural networks, such as glucose (Rouach et al., 2008) and lactate (Qu et al., 2000; Figley, 2011; Sotelo-Hitschfeld et al., 2015), and trophic factors essential for neuronal survival and differentiation (Gomes et al., 1999, 2005; Martinez and Gomes, 2002; Nones et al., 2012; Dezonne et al., 2013). Additionally, astrocytes maintain intimate contact along with endothelia and pericytes, thus contributing to the formation and function of the blood-brain barrier (BBB; Siqueira et al., 2018). More recently, a novel function has been attributed to astrocytes as part of the glymphatic system, in which astrocyte’s endfeet contact the vasculature, surrounding it and forming a system of tunnels, through which compounds such as glucose and amino acids are distributed, and the excess of toxic waste products is removed, such as large proteins including β-amyloid and tau (Weller et al., 2009; Iliff et al., 2012; Jessen et al., 2015).

**Figure 1:**
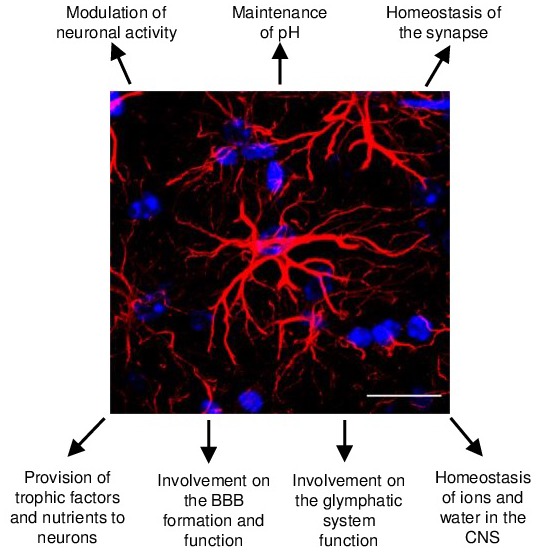
Schematic representation of the main astrocyte functions in the central nervous system (CNS). Astrocytes in the healthy murine CNS are highly ramified cells responsible for a number of functions in the CNS development, homeostasis and function. Glial fibrillary acidic protein (GFAP; in red) and DAPI (in blue) staining of murine hippocampus tissue. Scale bars, 20 μm.

Given the large number of functions performed by astrocytes (Figure 1), it is to be expected that deficits in these cells have a major impact on brain functioning. Such impact, however, due to the great diversity amongst these cells, vary among different brain areas and structures. This review article will focus on astrocyte diversity, highlighting the main findings on their morphological, functional and molecular differences amongst brain regions. We further discuss how such heterogeneity might contribute to shape the way astrocytes respond to different insults, such as those that rise from aging and in specific neurodegenerative diseases. Finally, we will argue that modulation of astrocyte phenotypes and functions, might shed light on new avenues to control aging and neural pathology.

## Molecular and Functional Heterogeneity of Astrocytes in the Healthy Brain

The neurocentric view of the brain has persisted for many years, and several studies have identified numerous neuronal populations that differ molecularly and functionally (Kepecs and Fishell, 2014; Jiang et al., 2015; Mancinelli and Lodato, 2018). Even though astrocytes are in close contact to neurons, it was not clear whether astrocytes would be as diverse as neurons. Astrocyte heterogeneity was an underexplored topic for many years, and just recently, the plurality of functions that they can exert in different neuronal circuits has shed a light on the fact that perhaps these cells have more specialized roles locally than previously thought.

In the mammal CNS, it has long been recognized a variety of astrocyte subtypes that differ regarding their developmental origin, morphology, physiology and metabolism amongst regions (Zhang and Barres, 2010; Oberheim et al., 2012). The first evidences of morphological heterogeneity of astrocytes were introduced by Golgi and Cajal (for review, Garcia-Lopez et al., 2010), and by William Lloyd Andriezen, World Health Organization (WHO), in 1893, described two different types of glial cells present in white and gray matter, the fibrous and protoplasmic glia, respectively (Andriezen, 1893), even though he thought these cells had different developmental origins. Since then, numerous evidence have corroborated the concept that protoplasmic and fibrous astrocytes are different subsets of astrocytes based on morphologic and molecular criteria (Vaughn and Pease, 1967; Raff et al., 1984; Raff, 1989). The protoplasmic astrocytes represent the biggest population of astrocytes in the gray matter, mainly found in the hippocampus and cerebral cortex; their cell bodies are extremely ramified, which probably allows them to touch numerous synapses typical of these regions, thus performing a neuromodulatory role (Bushong et al., 2002; Oberheim et al., 2012). The fibrous astrocytes are organized along white matter tracts; they are smaller and have lesser branching points than the protoplasmic astrocytes and can contact the nodes of Ranvier, thus contributing to keep the homeostasis in the region (Lundgaard et al., 2014).

Besides these two main subtypes of astrocytes, other astrocyte-like cells are described: radial glia cells (RG), initially described as merely playing a role in neuronal migration during cerebral cortex development, today RG are known as the main neuronal/glial progenitor present during brain development (Sild and Ruthazer, 2011); Bergmann glia, astrocyte type specific for cerebellum that ensheaths and controls cerebellar synapses, and is involved in granular cell migration (Rakic, 1971; Gregory et al., 1988; Grosche et al., 2002); Müller glia, astrocyte type specific for retina, involved in cell migration, neuronal generation and control of synapses of the retina (Reichenbach, 1989; Reichenbach and Bringmann, 2010). These specific subtypes of astrocytes and their heterogeneity will not be the scope of this review, but they provide an insight on how diverse the astrocyte population can be, especially as these subsets can vary amongst themselves.

The astrocytes that populate the human CNS are unique. Not only the cortical protoplasmic astrocytes are nearly three-fold larger in diameter, they also extend ten times more primary processes than their rodent counterparts and contact nearly 100-fold more synapses located in their territorial domains (Oberheim et al., 2009). The human brain also has exclusive subtypes of astrocytes, such as the *interlaminar astrocytes*, which cell body is located at the top cortical layer and sends long processes towards the deeper layers (Colombo et al., 1995) and the *varicose projection astrocytes*, which processes have many varicosities and extend 1–5 very long fibers towards all directions in the cerebral cortex (Oberheim et al., 2009, 2012). However, the whole extension of this diversity and its functional significance still remains to be investigated.

In rodents, by the end of gestation, RG-astrocyte differentiation is characterized, among several molecular mechanisms, by the replacement of RG markers, such as brain lipid binding protein (BLBP) and the intermediate filament (IF) protein, nestin, by astrocytic markers such as vimentin, the glial fibrillary acidic protein (GFAP), the glutamate transporter GLAST and the calcium binding protein S100β (Pixley and de Vellis, 1984) as discussed below. The correct timing of RG-astrocyte transformation is a crucial step to ensure correct number of neurons and cerebral cortex lamination. All things considered, the identification of astrocytic markers is crucial to the proper visualization of these cells, which may be the first step towards understanding how diverse they are. Although GFAP has become a classical marker for the identification of mature astrocytes, it has many limitations. One of the main ones is that it is not broadly expressed by all astrocytes across brain areas in the healthy CNS (Walz and Lang, 1998; Eng et al., 2000; Hol and Pekny, 2015).

GFAP has at least eight different isoforms generated by different splicing patterns, and they may be variably expressed in specific subsets of astrocytes (Middeldorp and Hol, 2011). Perhaps due to its importance in modulating astrocyte motility and shape by providing structural stability to astrocytic processes, GFAP expression is highly regulated including in injury and disease (Eng et al., 2000), and during aging in specific areas of the rodent brain, such as the corpus callosum, basal ganglia and hippocampus (Morgan et al., 1997, 1999) and in humans (Nichols et al., 1993). Another limitation is that GFAP has also been detected in peripheral glia, such as enteric glia (Kato et al., 1990), Schwann cells (Bianchini et al., 1992), and other non-neuronal cells, such as fibroblasts, and myoepithelial cells (Hainfellner et al., 2001). Other astrocytic markers include the glutamate transporters such as GLT-1 excitatory amino acid transporter 2 (EAAT2) and GLAST EAAT1, that stain a variety of astrocytes’ subtypes, e.g., RG, Bergmann glia, Müller cells, etc. (Schmitt et al., 1997; Williams et al., 2005), glutamine synthetase (GS) which stains a wide array of astrocytes’ subtypes in many regions where GFAP is not an effective marker (Anlauf and Derouiche, 2013); the glycoprotein S100β that is a more broadly expressed marker of astrocytes than GFAP in some regions (Ogata and Kosaka, 2002). However, these markers are less specific to astrocytes than GFAP, as S100β also stains oligodendrocytes and ependymal cells (Hachem et al., 2005; Steiner et al., 2007); GLT-1, GLAST and GS expression was also reported in neurons and oligodendrocytes (Cammer, 1990; D’Amelio et al., 1990; Schmitt et al., 2002). A few other markers have also been used to identify astrocytes such as aquaporin 4 (AQP4) and connexins 30 (Cx30) and Cx43, though these markers are mostly concentrated at astrocytes’ endfeet, rather than throughout the cell soma (Nagy et al., 1999; Nagelhus and Ottersen, 2013).

The advent of genetics era in recent decades and identification of the transcriptional profile of astrocytes, represented a new tool to study the diversity of the astroglial lineage and provided useful insights in the physiological state of these cells. The genetic profiling of astrocytes has identified a wider array of new markers such as the aldehyde dehydrogenase 1 family, member L1 (Aldh1L1) gene. This metabolic enzyme is responsible for the folate metabolism, which is especially important during neurulation. Aldh1L1 is widely expressed throughout the whole cell, and it seems to be expressed by most astrocytes, but not by other cell types (Cahoy et al., 2008), which might make it a better tool to visualize astrocytes in the developing mouse brain.

All markers identified so far, have showed some kind of specificity depending on the area: while Aldh1L1 was reported mainly staining cortical astrocytes (Waller et al., 2016); GS labeled mainly astrocytes in the entorhinal cortex (EC) comparing to GFAP (Anlauf and Derouiche, 2013), whereas astrocytes in the hippocampus are widely stained for GFAP (Bushong et al., 2002; Chai et al., 2017).

Altogether, although these data suggest that there is some kind of selectivity of astrocytic protein expression among different areas, the lack of a universal astrocyte marker or specific astrocytic regional markers still represents a challenge for the identification of intrinsic differences between astrocytes from distinct brain regions.

Recently, Chai et al. (2017) demonstrated that astrocytes from the murine striatum and hippocampus differ morphologically, functionally and molecularly. They found that even though the cell density in both regions were equally high, astrocytes in the striatum have a larger territorial size and contact nearly twice as many neuronal somas than hippocampal astrocytes, but the latter display stronger and more numerous physical interactions with excitatory synapses. At the functional level, these cells displayed different Ca^2+^-signaling dynamics and gap-junctional coupling. The transcriptomic and proteomic analysis of the astrocytes from these distinct regions showed that hippocampal and striatal astrocytes are molecularly distinct cell populations. Genes responsible for proteins with functional importance in astrocytes such as the ones encoding GLT-1, SPARC, the potassium channel inwardly rectifying potassium channel 4.1 (K_ir_4.1) and a sodium-dependent GABA transporter were amongst the 40 most highly expressed genes common between the hippocampus and striatum, but the most highly expressed gene in the hippocampal astrocyte was the one encoding GFAP, and in the striatal astrocyte was that encoding the protein μ-crystallin, a protein related to the regulation of the thyroid-hormone T3 (Vié et al., 1997), whose proper function in the brain is still unknown. Although these data corroborated the already known limitations of GFAP as an astrocyte marker, it suggests that μ-crystallin might be a molecular marker specific to striatal astrocytes (Francelle et al., 2015; Chai et al., 2017). Interestingly, they also found a gradient of expression of μ-crystallin within the striatum, suggesting that these cells may differ even depending on their subregion (Chai et al., 2017). Such differences have already been observed among astrocytes from the CA1 and CA3 subareas of the hippocampus demonstrating different physiological properties (D’Ambrosio et al., 1998), even though expressing a similar molecular marker profile (Sharif et al., 2004).

The functional implication of astrocyte heterogeneity in different neural circuits is still a matter of discussion. However, considering the key role of astrocytes in synapse formation, maturation and maintenance in different brain regions, it is likely that interactions between astrocytes and synapses vary accordingly to regional demands. Our group has recently compared the synaptogenic properties of astrocytes from four different brain regions: cerebral cortex, hippocampus, midbrain and cerebellum. We found that distinct populations of astrocytes have distinct synaptogenic profiles due to the differential expression of synaptogenic molecules such as TSP-1, glypicans 4 and 6, hevin, SPARC, TNF-α, brain-derived neurotrophic factor (BDNF) and TGF-β1 (Buosi et al., 2018). The synaptogenic properties of these astrocytes might reflect the differences in the requirement of astroglia’s synaptic coverage amongst different brain regions: some regions, like the cerebellum, have nearly all their synapses covered by astrocytes endfeet, whilst less than 50% of cortical and hippocampal synapses are tripartite synapses (Lippman et al., 2008; Witcher et al., 2010). Further, the pool of synaptogenic and anti-synaptogenic molecules produced by astrocytes might contribute to shape distinct neural circuits. In the cerebral cortex, for example, astrocytes can control the balance between excitatory and inhibitory synapses by activating different downstream signaling pathways, as described by our group. Astrocytes secrete TGF-β1, which induces the formation of either excitatory synapses *via* activation of D-serine production (Diniz et al., 2012), or inhibitory synapses *via* the Ca^2+^/Calmodulin-dependent protein kinase (CAM-kinase) pathway (Diniz et al., 2014b).

Together, data discussed here supports the idea that astrocytes display distinct inter- and intra-regional features, however, the functional significance of this is still a matter of discussion. The fact that astrocytic processes fill the local environment in non-overlapping domains suggest that a potential advantage of region-specified astrocytes might be their capacity to locally regulate neural circuit function (Emsley and Macklis, 2006; Oberheim et al., 2012). This raises a few unanswered questions: Can astrocyte heterogeneity primarily determine susceptibility of brain regions to different insults? Does astrocyte diversity impact the way brain regions age? Do astrocytes’ dysfunctions contribute equally to neurodegenerative diseases throughout the nervous system? In the next sections, we will discuss some recent advances and alternative explanations for the distinct vulnerability of these cells to different insults, diseases and aging amidst brain areas and the roles that astrocytes may play in these processes.

## Heterogeneity of Astrocyte’s Response to Diverse Insults: Reactive Astrocytes and Their Different Phenotypes

Astrocytes acquire different phenotypes in response to numerous pathological stimuli, such as stroke, neurodegenerative disorders, tumors, trauma, infection, ischemia and aging. These can trigger a response known as astrocyte reactivity, characterized by changes in the profile of astrocytes’ gene expression, leading to both morphological and functional changes that may vary from cellular hypertrophy, or atrophy, to proliferation and scar formation. The extent of the astrocytic reaction is severely impacted by the size of the affected area, the nature of the insult and its severity, the intensity of BBB disruption and the inflammatory response.

The first concept of astrocyte reactivity emerged with Virchow, who characterized a necrotic spinal lesion surrounded by a thick, highly fibrillary scarring, probably composed by densely packed astrocyte processes (Virchow, 1856). Even though astrocytes themselves were not formally described at that moment, accumulating pieces of evidence since then resulted in the classical hallmarks of reactive astrocytes: overexpression of GFAP, which frequently is related to the degree of reactivity, hypertrophy of cell body and increase in the number of cellular processes (Bignami and Dahl, 1976; Eng et al., 2000; Anderson et al., 2016). More recently, additional reactive astrocyte markers have been described, such as lipocalin-2 (Lcn-2), an acute phase protein (Lee et al., 2009), overexpressed by reactive astrocytes induced not only by ischemic stroke and lipopolysaccharide (LPS) exposure (Zamanian et al., 2012), but also by inflammation and excitotoxicity (Chia et al., 2011); and Serpin3n, a serine protease inhibitor (Zamanian et al., 2012), that was already reported to have its levels increased after injury in the rat facial and hypoglossal nuclei (Gesase and Kiyama, 2007). Identification of specific astrocytic reactivity markers is a crucial step towards the understanding of the unique mechanisms underlying this cellular process in different diseases.

Recently, the idea of astrocyte reactivity has been revisited. Rather than an all-or-none phenomenon, the process of becoming activated is gradated, and may vary from subtle changes to a deep irreversible morphological alteration, regulated in a context-dependent manner (Wilhelmsson et al., 2006; Sofroniew, 2009; Anderson et al., 2014). Further, although it is recognized that astrocytes can present gain or loss of function at the site of the insult, it is not clear whether this is beneficial or damaging to their surroundings (Sofroniew, 2009; Pekny et al., 2014). The reason for this controversy is due to the fact that apart from the protective roles that can be enhanced by astrocyte’s activation, such as production of anti-inflammatory factors, these cells can also acquire a toxic reactive phenotype, thus producing cytokines that exacerbate injuries in the spinal cord (Brambilla et al., 2009), increasing β-amyloid production (Nagele et al., 2003), or becoming atrophied and losing their neuroprotective functions in Alzheimer’s disease (AD), as will be discussed soon (Diniz et al., 2017).

Astrocytic reactivity is coordinated by complex molecular mechanisms, mainly initiated through an inflammatory response to the insults, in which gene expression and cellular changes are regulated by a series of inter- and intracellular signaling. Zamanian et al. (2012), was one of the first to describe reactive astrogliosis from a molecular standpoint. By using two injury models, a middle cerebral artery occlusion to induce ischemia and a systemic LPS injection, they found that reactive astrogliosis consisted of a rapid change in gene expression, but the reactive astrocyte phenotype varied according to the type of insult. Both models induced inflammation and caused concomitantly microglia activation and reactive astrogliosis, although by different mechanisms. They found that over 1,000 genes related to many different biological processes had their levels at least twice as high, when compared to quiescent astrocytes, and 260 of these were induced at least four-fold more in expression, hinting at the highly complex change that the astrocytic activation represents. They also found high levels of IF proteins such as GFAP and vimentin, and of many cytokines and their receptors, such as interleukin-6 (IL-6) and IL-1 receptor 1 (IL-1R1), confirming a few already described hallmarks of reactive gliosis. They found that the insults had overlapping but distinct sets of induced genes: from 263 reactive glial genes identified, 150 were preferentially expressed by ischemic stroke-reactive astrocytes, and 57 by LPS reactive astrocytes, and while ischemia induced a reactive astrocyte phenotype that was more protective, the insult with LPS produced a reactive astrocyte that was neurotoxic (Zamanian et al., 2012).

Recently, two different types of reactive astrocytes have been described in response to different insults (Liddelow et al., 2017), A1 and A2. In an LPS-induced neuroinflammation scenario, activated microglia induced a neurotoxic reactive astrocyte phenotype by secreting IL-1α, TNFα and complement component 1q (C1q). The so-called A1 astrocyte showed loss of normal functions and gain of new, harmful, functions at the same time. They upregulate many of the markers mentioned previously on this review article, such as LCN-2, Serpina3, and the classical GFAP. Liddelow et al. (2017) have demonstrated that the A1 astrocyte loses normal functions, such as the ability to induce synapse formation and this could be due to the increased expression of many classical complement cascade genes that are toxic to synapses, even though the specific molecule toxic to neurons remains unknown.

On the other hand, other insults, such as ischemia, can induce a protective reactive astrocyte. In this case, reactive astrocytes may secrete neuroprotective molecules, such as TSPs that upregulate many neurotrophic factors, and promote CNS recovery and repair (Papadopoulos et al., 2004; Zador et al., 2009). This second phenotype is called A2 astrocytes, and they are known to secrete neuroprotective cytokines.

We previously demonstrated that, in a sepsis model, activated astrocytes were observed both in the hippocampus and cerebral cortex, whilst activated microglia was mainly observed in the hippocampus; moreover, both of these areas showed impairment of synapse function (Moraes et al., 2015). To further investigate the molecular effects of sepsis on these cell populations, we have used stimulation with LPS to mimic the inflammatory effect observed during sepsis. As result, LPS stimulation of glial cells generated distinct secretory profiles in astrocytes and microglia, which had direct, though contrasting, impacts on synapses. The conditioned medium of LPS-stimulated microglia induced synaptic elimination, whilst the medium of astrocytes treated with LPS increased synapse number. Both cell types showed increased production of TNFα and IL-6, and while astrocytes had increased production of TGF-β1, an anti-inflammatory and synaptogenic cytokine, microglia showed elevated secretion of IL-1β, a pro-inflammatory cytokine, especially in the hippocampus (Moraes et al., 2015). These data highlight different responses of glial cells under an insult; whereas LPS-activated microglia present a neurotoxic phenotype; LPS-activated astrocytes, presented a neuroprotective role, similar to those called A2 astrocytes.

The knowledge of how these cells act in the healthy brain just adds another layer of complexity to the immense possibilities of phenotypes that they can acquire after an insult or aging. It is theorized that there is a gradient between the A1, neurotoxic phenotype and the A2, neuroprotective phenotype, and, possibly, an interplay between different profiles (Liddelow and Barres, 2017). It is imperative to consider the diversity of astrocyte’s roles and their heterogeneity in health in order to understand more deeply their roles in disease and aging, as we will discuss in the next sections.

## Aging and Neurodegenerative Diseases: Impact on Astrocyte Reactive Phenotypes

The concept of astrocyte heterogeneity has profoundly changed our view on astrocyte functions, astrocyte-neuron interactions as well as their impact in aging and neurodegenerative diseases. The first evidence on astrocyte heterogeneity referred to their inter and intra-regional diversity in morphology in the CNS (Oberheim et al., 2009). Moreover, besides morphology, astrocytes are highly diverse in terms of molecular expression pattern and functions, as discussed in the previous sections.

Emerging lines of evidences have shown that global features of astrocytic reactivity, such as changes in astrocyte morphology, transcriptional profile and function, are distinctly observed in specific brain regions during early stages of many neurodegenerative diseases, such as AD, Parkinson’s disease (PD), Huntington’s disease (HD), and amyotrophic lateral sclerosis (ALS; Phatnani and Maniatis, 2015). These observations raise two main questions: “Is astrocyte heterogeneity maintained throughout the aging and in neural diseases?” and “How might astrocyte heterogeneity affect the onset and progression of age-related cognitive decline and age-related diseases?” The next sections will shed light on the possible involvement of changes in astrocyte phenotype to brain aging and pathology.

### Aging

Astrocyte reactivity is also a hallmark of physiological aging in rodents, non-human and human primates (Nichols et al., 1993; Rodríguez et al., 2014; Robillard et al., 2016). It is interesting to note that similarly to some neurodegenerative diseases, during aging, astrocyte reactivity is mainly observed in specific brain regions primarily targets for synaptic loss and age-related cognitive decline, such as the hippocampus and frontal cortex (FC; Rodríguez et al., 2016). Nevertheless, the actual contribution of astrocytes to regional vulnerability of the nervous system to specific diseases remains to be investigated.

Interestingly, it has also been reported an intra-regional heterogeneity in astrocyte’s response to brain aging. Astrocytes in the hippocampal dentate gyrus and CA1 presented an age-dependent hypertrophy, as observed by a reorganization of the cytoskeleton protein, GFAP, leading to an increased surface, volume and somata volume of astrocytes in aged mice; while the opposite effect was observed in the EC. On the other hand, S100β-immunostaining, which is a cytosolic protein and hence shows a more complete astrocyte arborization than GFAP, revealed an increased surface area and volume in aged astrocytes in the EC and dentate gyrus, but not in CA1. Therefore, these observations imply that morphological changes based only on GFAP-staining may not reflect the entire complexity of astrocytes, as observed in the EC, and hence it may cause misinterpretations. In conclusion, this study suggested that astrocytes undergo complex region-specific morphological and molecular changes upon aging (Rodríguez et al., 2014).

In agreement with these results, an age-dependent increase in hippocampal GFAP expression has also been shown by other groups in rodents (Hayakawa et al., 2007; Lynch et al., 2010; Cerbai et al., 2012) and most prominently in the human hippocampal formation (David et al., 1997), but also in the FC and temporal cortex (TC; Nichols et al., 1993). Nevertheless, functional implication of GFAP upregulation during aging is still a matter of discussion, as well as how and why particularly hippocampal astrocytes undergo these changes.

Within this context, several studies have been trying to elucidate the transcription profile of astrocytes from different brain regions during aging, taking advantage of new techniques for the isolation of purified cell populations from the mouse brain or post-mortem human brain tissues. These new approaches, combined with transcriptome analysis, have shed light on the molecular signature of aged astrocytes as well as their region-specific changes during the aging process.

The physiological aging is characterized by a chronic, low-grade and systemic inflammation, referred as “inflammaging”, which is an important risk factor for morbidity and mortality in elderly people (Franceschi et al., 2000). It is also known that glial cells, specially microglia and astrocytes, play a preponderant role in controlling neuroinflammation. As previously discussed, glial cells undergo morphological, molecular and functional changes that will result in pro or anti-inflammatory phenotypes, depending on the pathological context and age (Colombo and Farina, 2016). However, only recently our knowledge on the role of astrocytes in inflammaging and their regional differences in response to the aging process have beginning to be better elucidated.

Orre et al. (2014) showed that astrocytes isolated from the aged mouse cerebral cortex present increased inflammatory phenotype, although decreased GFAP expression compared to young astrocytes. It is noteworthy that although an upregulation of GFAP is usually associated with astrocyte reactivity, this feature itself may not be entirely precise to characterize this process, either in aging or brain disease. This is due to several reasons, such as: the number of GFAP positive cells and the basal GFAP expression level notably vary between different brain regions (Kimelberg, 2004); alteration in GFAP expression is usually region-specific (Rodríguez et al., 2014) and it may depends on the type and stage of the disease, or even the time after injury (Kamphuis et al., 2014; Diniz et al., 2017; Cunha et al., 2018).

Supporting astrocyte heterogeneity in aging, evidence have shown that astrocytes from distinct brain regions present an unique molecular signature in both aged mouse and human brain tissue (Soreq et al., 2017; Boisvert et al., 2018; Clarke et al., 2018). Boisvert et al. (2018) performed RNA-seq on astrocytes isolated from four brain regions of adult and aged mice: motor and visual cortex, hypothalamus and cerebellum. Surprisingly, they found significantly more changes in these cells from the hypothalamus and cerebellum, than from the cortex. They found increased expression of genes for inflammatory response and astrocyte reactivity, including GFAP and Serpin3n, and synapse elimination pathways, mainly represented by proteins of the complement system, such as complement component 3 (C3) and complement component 4b (C4b), and decreased cholesterol synthesis enzymes (Boisvert et al., 2018).

Similarly, Clarke et al. (2018) compared the expression profile of astrocytes isolated from the hippocampus, cortex and striatum of young and aged mice. They observed that hippocampal and striatal astrocytes showed more pronounced changes compared to cortical astrocytes. Corroborating previous data, they observed the most prominent class of upregulated genes upon aging was related to astrocyte reactivity, immune response and synapse elimination (Clarke et al., 2018).

Therefore, these studies have suggested that murine astrocytes undergo age-dependent changes in gene expression that may contribute to age-related synapse loss and neuroinflammation. Further, they reinforce the region-specificity of astrocyte responses to the aging process. Nevertheless, there is still a lack of evidence concerning the functional implications of these changes in gene expression to astrocyte function and cellular interplays in aging. Moreover, whether human astrocytes behave similarly to their murine counterparts is still a matter of discussion.

Within this context, Soreq and collaborators reported the first evidence of glial region-specific gene expression in the human brain aging, by analyzing ten different brain regions from the post-mortem human tissue of individuals aged from 16 to 102 years. They demonstrated that while a general upregulation of microglial and endothelial genes was observed upon aging in all brain regions analyzed; astrocyte- and oligodendrocyte-specific genes showed a more complex shift in their expression pattern, prominently observed in the hippocampus and substantia nigra (SN). On the other hand, these changes were not evident for the neuron-specific genes (Soreq et al., 2017). Altogether, these data might suggest another view on the concept of “selective vulnerability” in aging and age-related diseases; a concept that primarily refers to an increased vulnerability of specific brain regions or groups of cells to a pathological state or injury. The vulnerability of neuronal populations has been mainly studied and characterized in neurodegenerative diseases, as reviewed by other works (Saxena and Caroni, 2011; Fu et al., 2018). However, recently new evidence has pointed that major changes in the transcriptional profile and function of glial cells might also contribute to predict the human brain aging and to understand the selective region-specific vulnerability in these age-related neurodegenerative diseases.

### Neurodegenerative Diseases

During the last decades, a growing number of evidence has suggested that astrocytes and microglia play a key role in synaptic dysfunction, neuronal loss and cognitive impairments associated with several neurodegenerative diseases, such as AD; ALS, HD and PD. Interestingly, changes in astrocytes arise early in the course of these conditions and most prominently in specific brain regions known to be primarily vulnerable to a particular pathology. However, the cellular and molecular basis that define the selective vulnerability of specific regions in neurodegenerative diseases remain poorly understood. Understanding astrocyte heterogeneity in this context certainly represents an important step to unveil the mechanisms underlying the onset and progression of neurodegenerative diseases.

In this section, we will discuss recent studies on how astrocytes’ changes and heterogeneity might impact in the onset of two neurodegenerative diseases, AD and PD.

#### Alzheimer’s Disease

AD is the most common cause of dementia and one of the main public health concerns nowadays (Scheltens et al., 2016). According to the WHO a considerable increase in incidence of dementia and age-related diseases, especially AD, is expected for the next few years. Within this context, many efforts have been done to better elucidate the mechanisms involved in AD pathology and unveil possible cellular and molecular targets for diagnosis, prevention and/or therapy.

Clinically, AD is characterized by cognitive deficits, mainly represented by learning impairments and memory loss. Histopathologically, the two major hallmarks of AD are extracellular amyloid plaques (APs) depositions, composed of amyloid β (Aβ) peptides, and intracellular neurofibrillary tangles, constituted by hyperphosphorylated Tau protein (Ballard et al., 2011). Initially, it was thought that Aβ deposition would be the primary event in AD pathology, triggering neurofibrillary tangle formation and neuronal death, which led to the “amyloid cascade hypothesis” (Hardy and Higgins, 1992). However, it has been demonstrated that the most neurotoxic forms of Aβ are their soluble ligands, Aβ oligomers (AβOs), shedding light on the “Aβ oligomer hypothesis” (Lambert et al., 1998).

AβOs accumulate at early stages in AD pathology, before plaque formation, and clinical symptoms, in humans and animal models (Gong et al., 2003; Noguchi et al., 2009; Pham et al., 2010; Lesné et al., 2013). Currently, it is well known that AβOs induce memory impairment, disruption of long-term potentiation (LTP) and long-term depression (LTD; Townsend et al., 2006; Shankar et al., 2008), synapse dysfunction and loss (Brito-Moreira et al., 2017; Diniz et al., 2017), oxidative stress (Sponne et al., 2003; De Felice et al., 2007), endoplasmic reticulum (ER) stress (Umeda et al., 2011; Lourenco et al., 2013) and neuroinflammation (Forny-Germano et al., 2014).

AD pathology initiates in a region-specific manner, meaning that specific brain regions or group of cells are more vulnerable to AD than others. The selective vulnerability of neuronal populations has been better elucidated and reported in the EC, subiculum and the hippocampal CA1 region (Reilly et al., 2003; Stranahan and Mattson, 2010), but also in the basal forebrain (Whitehouse et al., 1982) and locus coeruleus (Bondareff et al., 1987). For a more complete view on neuronal selective vulnerability we recommend additional review (Fu et al., 2018). Besides neuronal contribution, several evidences have shown that glial changes are also present early, before neuronal death and clinical symptoms, during the pathogenesis of AD.

**Figure 2:**
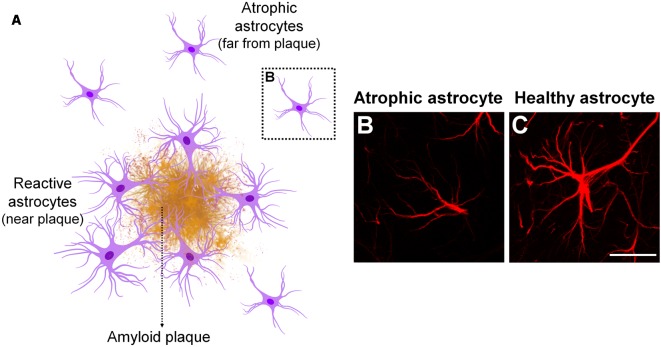
Astrocyte phenotype in Alzheimer’s disease (AD). In AD pathology, morphological changes in astrocytes depend on the stage of disease and proximity to amyloid plaques (APs; **A**). Astrocyte atrophy occurs at early stages of AD, before plaque formation, as well as in non-plaque associated astrocytes at later stages of disease **(B)**. Atrophic astrocytes present reduced number and thinner cellular processes, which is accompanied by diminished volume and somata volume, compared to healthy or resting astrocytes **(C)**. Astrocyte hypertrophy is mainly observed in astrocytes surrounding APs at later stages of AD. They show opposite features in comparison to atrophic astrocytes.

The likely involvement of astrocytes in AD pathology has been already reported by Alzheimer (1910), through the observation of association between glial cells and senile plaques in demented brains (Alzheimer, 1910; Verkhratsky et al., 2010). Subsequent studies have shown that astrocytes undergo complex morphological, transcriptional and functional alterations in AD. Morphologically, astrocytes may become either atrophic or hypertrophic depending on the stage of the disease and proximity to APs depositions (Rodríguez-Arellano et al., 2016; Figure 2).

Astrocyte atrophy has been observed during early stages of AD pathology as well as in non-plaque associated astrocytes along disease progression in the EC, medial prefrontal cortex, dentate gyrus and CA1 hippocampal region in 3xTg-AD mouse model (Olabarria et al., 2010; Kulijewicz-Nawrot et al., 2012; Yeh, 2013), PDAPP-J20 transgenic mice (Beauquis et al., 2013), and in CA1 of adult mice intracerebroventricularly injected with AβOs (Diniz et al., 2017). The functional meaning of this observation is still a matter of discussion. A possibility is that a reduction in astrocyte territorial domains might result in diminished synaptic coverage and impaired neuro-vascular unit; two important features that might be involved in synaptic dysfunction and loss of metabolic support at early stages of AD. Nevertheless, the underlying mechanisms of astrocyte atrophy as well as the functional impact of that to the onset of AD pathology have not been completely elucidated so far.

Within this context, recent data from our group have reinforced that astrocytes are targets for AβOs and that these neurotoxins induce molecular and functional changes in these cells, leading to astrocyte activation and impairments in their synaptogenic and synaptical-protective capacity (Diniz et al., 2017). We showed that AβOs rapidly interacted with astrocytes and triggered astrocyte activation *in vitro*. Further, the intracerebroventricular injection of AβOs in adult mice acutely induced astrocyte atrophy in the CA1 hippocampal region, as observed by reduced number of primary processes, area and intensity of GFAP per cell. Following these changes, we verified that AβOs reduced the levels of TGF-β1 in astrocytes, an event that was related to an impairment in the synaptogenic and neuroprotective potential of astrocytes (Diniz et al., 2017). These data together with the fact that TGF-β1 is an important cytokine that promotes inhibitory and excitatory synapse formation in the CNS (Diniz et al., 2012, 2014b; Araujo et al., 2016), shed light on a new possible mechanism involved in synapse dysfunction and loss at early stages of AD pathology, mediated by astrocytes.

In contrast, many evidences have shown that during later stages of AD, astrocytes nearby plaques become highly hypertrophic and produce increased levels of GFAP (Olabarria et al., 2010; Heneka et al., 2015). Such features have also been observed in post-mortem human brain tissue, which seems to accompany the Braak stage progression (Simpson et al., 2010; Kamphuis et al., 2014). In addition, a transcriptomic analysis of astrocytes isolated from aged APPswe/PS1dE9 transgenic AD mice revealed that these cells acquired a pro-inflammatory phenotype, as well as a less supportive capacity to neuronal communication, represented by reduced expression of genes involved in that process (Orre et al., 2014). Altogether, these evidences suggest that the reactive phenotype of astrocytes in AD might contribute to neuroinflammation, synaptic deficits and neuronal dysfunctions during disease progression. Interestingly, a recent study has corroborated that hypothesis through the genetic inhibition of astrocyte reactivity in an AD mouse model. Ceyzériat et al. (2018) have shown that the inhibition of the astrocytic janus kinase 2 (JAK2)/signal transducer and activator of transcription 3 (STAT3) pathway efficiently controlled the reactivity of these cells in the hippocampus of AD mice, which was followed by a reduction in amyloid deposition, synaptic deficits and spatial learning improvements.

Therefore, altogether, these data strongly point to the key involvement of reactive astrocytes in the progression of AD pathology, revealing their detrimental actions on neuronal communication and cognitive performance. Nevertheless, apart from being the two major hallmarks of astrocyte reactivity, it remains elusive the functional meaning of astrocyte hypertrophy and upregulation of GFAP levels at later stages of AD.

Furthermore, since these alterations are mostly reported in vulnerable brain regions to AD pathology, in which neuronal degeneration is mainly observed, it is possible that astrocyte heterogeneity plays a role in this selective vulnerability. It has been suggested that the pre-clinical stage of AD begins decades before clinical symptoms, which is called the cellular phase of AD pathology (De Strooper and Karran, 2016). During this phase, extensive changes occur in glial cells and vasculature, which may orchestrate subsequent neuronal deficits. In fact, Soreq et al. (2017) have found that age-related changes in gene expression profile of astrocytes take place particularly in the hippocampus and SN in human tissue, two regions primarily affected during the pathogenesis of AD and PD. Therefore, these observations strengthen the likely involvement of astrocyte heterogeneity to the regional selective vulnerability in AD onset and progression. However, a direct link between astrocytes morphological/molecular changes to functional alterations still remains to be determined.

#### Parkinson’s Disease

PD is the second most common type of neurodegenerative disease, affecting 2%–3% of the elderly population. Similarly to AD, the majority of PD cases is sporadic, while less than 10% are associated to genetic causes (Poewe et al., 2017). Clinically, PD is primarily characterized by motor abnormalities, mainly represented by tremor at rest, rigidity, bradykinesia and postural instability (Jankovic, 2008), symptoms that have been linked to a progressive and selective degeneration of dopaminergic neurons in SN pars compacta (SNpc) during early stages of the disease (Dauer and Przedborski, 2003). However, several other CNS and peripheral nervous system (PNS) structures are also affected early or during the disease progression, a fact that is responsible for the non-motor symptoms of PD, which include, for example, autonomic dysfunction, olfactory deficits and cognitive decline (Schapira et al., 2017).

Histopathologically, aggregates of the synaptic protein α-synuclein are usually found in the cell bodies and neurites, called respectively Lewy bodies and Lewy neurites, in specific classes of neurons in several brain regions in all PD patients (Poewe et al., 2017). Although it is still debated, Braak et al. (2003) have proposed that Lewy bodies’ pathology spread from one region to another following a specific and predictive progression during the course of PD. Interestingly, more recently it has been suggested a prion-like propagation of α-synuclein aggregates. In this hypothesis, α-synuclein might be transmitted from neuron to neuron, along axons, secreted into the extracellular space and internalized by neighboring cells, including neurons and glial cells. Once internalized, it may induce the aggregation of the native proteins in the host, contributing and exacerbating the aggregate spreading (Brundin et al., 2010). Although this hypothesis has been proven in both *in vitro* and *in vivo* models (Desplats et al., 2009; Hansen et al., 2011), it remains to be determined whether this mechanism explains the spreading of Lewy’s body pathology in humans.

Several lines of evidence have shown that impairments in α-synuclein proteostasis strongly contribute to PD pathogenesis, as well as may impact other cellular pathways and functions also implicated in PD, such as mitochondria dysfunction, oxidative stress and neuroinflammation (Poewe et al., 2017). Moreover, astrocytes and microglia seem to play an important role in all of these pathological mechanisms, contributing to PD onset and progression (Halliday and Stevens, 2011).

Astrocytes accumulate α-synuclein aggregates in the SN and extra nigral regions in patients with PD, which is correlated with the severity of neuronal loss (Wakabayashi et al., 2000). Interestingly, it has also been reported that particularly protoplasmic astrocytes presented inclusions of protein aggregates in PD, which was not observed in fibrous astrocytes (Song et al., 2009). Braak et al. (2007) have shown that α-synuclein immunoreactive astrocytes are present in several brain regions of PD patients, which appears to accompany the formation of Lewy bodies and neurites. Moreover, these immunoreactive astrocytes were also found in the striatum and dorsal thalamus, regions where Lewy’s bodies do not develop, suggesting that astrocytes might internalize α-synuclein released by dysfunctional axon terminals in these regions. This is supported by observations that astrocytes can take up α-synuclein released by neurons, which, in turns, induce gene expression changes in astrocytes, mainly represented by an up-regulation of pro-inflammatory genes (Lee et al., 2010). Conversely, an *in vitro* study has proposed that human astrocytes are similarly able to transfer aggregated α-synuclein to neighboring astrocytes, *via* direct contact and tunneling nanotubes (TNTs; Rostami et al., 2017). Moreover, accumulation of α-synuclein in astrocytes may impair their lysosomal-autophagosomal machinery as well as lead to ER swelling and mitochondria damage (Rostami et al., 2017). Nevertheless, it remains to be investigated whether these events also occur *in vivo* and their overall impact to the aggregate spreading in PD pathology.

The direct functional impact of non-cell autonomous mechanisms on neurodegeneration in PD has been reported in mouse models in which solely astrocytes expressed PD-related A53T α-synuclein mutation. In this case, α-synuclein aggregation in astrocytes resulted in severe astrogliosis, disruption in the BBB and down-regulation of glutamate transporters. Moreover, microglial activation was also pronounced especially in the brainstem and SN, in which neuronal loss occurred (Gu et al., 2010). Additional studies have unraveled the molecular mechanisms underlying astrocyte and microglia activation in PD. The treatment of astroglial and microglial cell cultures with recombinant α-synuclein induced cell activation, as observed by a strong inflammatory response and production of reactive oxygen species (ROS), effects dependent on Toll-like receptor 4 (TLR4) activation in both cell types. Further, the ablation of TLR4 in α-synuclein-treated microglia and astrocytes suppressed their pro-inflammatory response, although the uptake of α-synuclein by astrocytes was independent of TLR4 (Fellner et al., 2013; Rannikko et al., 2015). These studies have shed light on new signaling pathways involved in α-synuclein-induced neuroinflammation in PD, as well as the key role of glial cells in disease progression.

Although the underlying mechanisms involved in PD pathology have been extensively investigated, there is still a lack of evidence concerning the cellular and molecular determinants of regional selective vulnerability in PD. Curiously, as discussed above, astrocytes changes are largely present in regions primarily affected during the onset of PD in humans (Wakabayashi et al., 2000; Braak et al., 2007); further, the non-cell autonomous mechanisms of dopaminergic neuronal loss have also been proved in PD animal models (Gu et al., 2010; Booth et al., 2017). However, although these evidences point to the likely involvement of astrocytes in PD, they still do not explain why degeneration begins at specific brain regions. Within this context, new studies on the inter-regional molecular heterogeneity of astrocytes have shed light on the role of these cells in predicting the human brain aging, with implications to age-related diseases, such as PD.

Notably, it has been shown that the main age-related shifts in gene expression profile of astrocytes occur in the hippocampus and SN from post-mortem human tissue (Soreq et al., 2017). Since the aging process is a risk factor for PD, it is possible that the glial alterations in SN along aging may play a role in determining the selective vulnerability of this region to PD.

Recently, an outstanding study has strengthened the role of astrocyte-microglia interplay in PD pathology. It is well known that the state of microglia activation may have a direct impact on the astrocyte phenotype in different pathologies (Liddelow and Barres, 2017; Shinozaki et al., 2017). Recently, this hypothesis was strongly demonstrated for PD. By using the agonist of the glucagon-like peptide-1 receptor (GLP1R), NLY01, the authors control the pro-inflammatory profile of microglia treated with α-synuclein preformed fibrils (α-syn PFF) *in vitro*. NLY01 pre-treatment indirectly controls astrocyte reactivity by reducing microglial secretion of molecules involved in astrogliosis, such as IL-1β, TNF-α and C1q. Similarly, astrocyte reactivity in the ventral midbrain was inhibited in PD mice pre-treated with NLY01. These effects were accompanied by a prevention of dopaminergic neuronal loss, reduced dopamine levels and motor deficits in PD mice (Yun et al., 2018).

Several evidences have highlighted the key involvement of glial cells in neurodegenerative diseases. However, many unanswered questions remain concerning the contribution of glial cells to the selective vulnerability of the nervous system to these diseases. Altogether, elucidating the molecular and functional heterogeneity of astrocytes arise as a fundamental step to understand the underlying mechanisms of age-related cognitive decline and associated neurodegenerative diseases. Further, it might reveal new therapeutic targets to modulate astrocyte phenotype in order to prevent and/or delay these processes.

## Future Directions: Glial Cells as Novel Targets for Therapeutic Strategies

**Figure 3:**
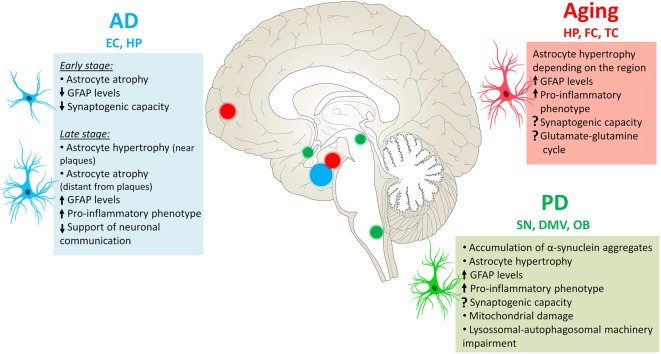
Astrocytes’ changes in aging and neurodegenerative diseases. During the physiological aging, astrocytes in the hippocampus (HP), frontal cortex (FC) and temporal cortex (TC) become reactive, with increased levels of GFAP and pro-inflammatory phenotype. In AD, astrocyte reactivity is mainly observed in the entorhinal cortex (EC) and HP, where these cells can be either atrophic or hypertrophic depending on the stage of the disease and their proximity to AP. In Parkinson’s disease (PD), astrocytes accumulate α-synuclein aggregates in several CNS regions, particularly in the substantia nigra (SN) and extra-nigral regions affected in PD pathology, such as the dorsal motor nucleus of the vagus (DMV) and olfactory bulb (OB). The functional changes exposed here are not necessarily observed in all mentioned regions, more information can be found in the text.

Astrocyte reactivity is a common feature in aging and age-related diseases. As discussed in this review article, although its functional implications are not completely understood yet, new evidence have suggested that reactive astrocytes comprise a heterogeneous group constituted by subpopulations of astrocytes that may differ from each other in terms of molecular signature, function and response to several physiological and pathological stimuli. These cells can undergo atrophy and hypertrophy amongst other changes according to the insult (Figure 3), therefore altering their functions in the region.

Differently from previously thought, astrocyte reactivity is not just a hallmark of aging and brain diseases, but a key mechanism involved in the pathogenesis and progression of these conditions. It is worth noting that, as there is a crosstalk between microglia and astrocytes, these cells can greatly influence each other’s phenotype (for review see Jha et al., 2018), therefore, by modulating microglia phenotypes, it would be possible to change the way astrocytes respond to injury and disease as well. As described before, microglia can respond to inflammatory insults by becoming activated and influence astrocyte’s response by secreting cytokines and complement cascade molecules such as TNF-α and C1q (Liddelow et al., 2017), and during aging, extensive neuroinflammation is also observed (Franceschi et al., 2000). Therefore, as it can determine the fate of astrocytes, it is important to consider microglia when designing new drugs that could modulate astrocyte’s phenotypes, especially as the modulation of microglia towards an anti-inflammatory phenotype may provide neuroprotective effects, contributing to the recovery of the diseased brain (Cherry et al., 2014; Song and Suk, 2017). Suk (2017), suggested that more importantly than blocking glial cells reactivity, the focus should be on new strategies that could modulate glial cells activated phenotype.

Thus, modulation of astrocyte reactivity state emerged as an important venue for the development of new preventive and therapeutic strategies (Yun et al., 2018). Within this context, molecules and compounds, including natural or synthetics, able to control astrocyte reactivity and function have been viewed as potential drugs to neurodegenerative diseases.

Within the natural compounds, the biggest class of polyphenols in nature and also in the human diet comprises the flavonoids. These molecules are ubiquitously found in vegetables, cereals, fruits, tea, chocolate and wine (Tsao, 2010). Epidemiological studies have shown that the long-term consumption of polyphenols is associated to a reduced risk in developing cardiovascular diseases, diabetes, cancer, and neurodegenerative diseases (Arts and Hollman, 2005; Graf et al., 2005).

The consumption of polyphenol-rich diets has been linked to an enhanced cognitive performance and delay in age-related cognitive decline in humans (Devore et al., 2012; Brickman et al., 2014). In agreement, the administration of flavonoids or polyphenol-rich extracts may promote learning and memory improvements in aged animals (Zeng et al., 2012; Bensalem et al., 2018), as well as in several mouse models for neurodegenerative diseases, including AD (Zhang et al., 2014), ALS (Koh et al., 2006) and PD (Rojas et al., 2012), for review see (Solanki et al., 2015). However, the cellular and molecular mechanisms underlying the flavonoids actions are still not fully understood. Besides their remarkable antioxidant effect, flavonoids can also directly interact with cellular receptors as well as modulate signaling pathways in neurons and glial cells (Williams and Spencer, 2012).

Due to the prominent involvement of astrocyte reactivity/inflammation in aging and neurodegenerative diseases, a growing number of studies has pointed that these cells may be important targets for the natural compounds in the CNS (Matias et al., 2016). Strikingly, flavonoids have been also reported to modulate astrocyte reactivity and hence attenuating neuroinflammation. It has been shown that the flavonoids luteolin and quercetin were able to inhibit IL-1β-induced astrocyte activation, which was characterized by a reduced secretion of pro-inflammatory cytokines and chemokines, such as IL-6, IL-8, IP-10, MCP-1, an up-regulation of antioxidant enzymes, SOD1 and thioredoxin (TRX1), as well as an down-regulation of the GFAP levels in astrocytes (Sharma et al., 2007). In agreement, Bahia et al. (2008) demonstrated that the flavonoid (−) epicatechin specifically induced the activation of the antioxidant response element (ARE) pathway in astrocytes, which was followed by an increase in the glutathione (GSH) levels, an important antioxidant enzyme.

Furthermore, the pre-treatment of glial cell cultures (astrocytes and microglia) with the flavonoids naringenin, hesperidin, (+)-catechin or (−)-epicatechin significantly attenuated the production of TNF-α by these cells when exposed to LPS/interferon-γ (IFN-γ). Most prominently, naringenin not only reduced glial cell activation, but also promoted neuronal survival in a co-culture system with neurons and glia (Vafeiadou et al., 2009). Altogether, these studies have pointed that the modulation of astrocyte reactivity may have an indirect beneficial effect on neuronal physiology.

Further effects of flavonoids in controlling astrocyte reactivity have also been suggested by other groups in animal models of aging and neurodegenerative diseases. Aged rats treated with the polyphenol resveratrol showed increased neurogenesis and microvasculature and reduced astrocyte hypertrophy, as well as more ramified microglia (non-activated) within different hippocampal regions. These effects were accompanied by improvement in memory performance (Kodali et al., 2015).

In APPswe/PS1dE9 double transgenic AD mouse model, the long-term oral administration of fisetin significantly reduced astrocyte reactivity, mainly observed by a reversal of astrocyte hypertrophy and a down-regulation of the GFAP levels in AD mice (Currais et al., 2014). Similar results have also been observed in p25Tg AD mouse model treated with the phenolic compound Curcumin (Sundaram et al., 2017).

Data from our group have recently suggested beneficial effects of flavonoids also in the healthy brain. We have shown that hesperidin and casticin increased the neuroprotective potential of astrocytes *in vitro*, by modulating the secretion of protective factors (de Sampaio e Spohr et al., 2010; Nones et al., 2012). Although the identity of those factors is not fully characterized, further studies have suggested a number of neurotrophic factors that might be target for the flavonoids’ actions. A screening of thirty-three different flavonoids revealed that calycosin, isorhamnetin, luteolin, and genistein were more promising in inducing the synthesis and secretion of the nerve growth factor (NGF), glial-derived neurotrophic factor (GDNF), and BDNF by astrocytes in culture (Xu et al., 2013).

More recently, our group raised evidences of the beneficial effects of the flavonoids in the healthy brain *in vivo*. The treatment of adult mice with the flavanone hesperidin induced improvements in memory performance, which were accompanied by increased synaptic density in the hippocampal CA1 region, as well as activation of the TGF-β pathway in the same region. We also showed that hesperidin was able to enhance the synaptogenic potential of astrocytes, by modulating the TGF-β1 pathway and levels (Matias et al., 2017), a pathway previously involved in the regulation of synapse formation in the CNS (Diniz et al., 2014a). Deficits in this pathway is implicated in neurodegenerative diseases, including AD (Diniz et al., 2017, 2018). Whether the effect of hesperidin/TGF-β1 in astrocytes and thus in cognitive decline is related to their anti-inflammatory activities or not, remains to be elucidated. Nevertheless, modulation of the astrocytic TGF-β1 pathway might arise as a valuable strategy in promoting cognitive improvements in both physiological and pathological contexts. Based on that, targeting of specific signaling pathways that modulates astrocyte phenotypes emerged as a promise therapeutic strategy.

Recently, modulation of the JAK/STAT3 pathway has been shown as a useful strategy to control astrocyte reactivity *in vivo*. By specifically inhibiting the astrocytic JAK/STAT3 activation in an AD mouse model, Ceyzériat et al. (2018) reduced astrocytes reactivity and amyloid deposition, thus leading to improvements in their cognitive performance. Similarly, as previously discussed, block of A1 astrocyte conversion by microglia by the agonist (NLY01) of the GLP1R has proven to be neuroprotective in models of PD. The treatment of PD mouse models with NLY01 significantly prevented the dopaminergic neuronal loss in the ventral midbrain, restored dopamine levels as well as ameliorated the motor deficits observed in PD mice (Yun et al., 2018). Despite all the new advances, there are still many unanswered questions in this field, such as “Is it possible to convert a neurotoxic reactive astrocyte in a neuroprotective one?”; “Are the human astrocytes more diverse in terms of reactive phenotypes?”; “Are the mechanisms involved in the induction of A1 and A2 astrocytes similar in different species, such as rodents and humans?”.

In addition, due to the large genetic variability in human population and the fact that human astrocytes are unique in terms of morphological and functional complexity (Colombo et al., 1995; Oberheim et al., 2009), new studies are needed to investigate whether our knowledge on murine astrocytes can be applied to their human counterparts. Interestingly, a new study has shown that changes in astrocyte gene signature may have functional implications to the age-related cognitive decline as well as may predict the human aging with greater precision than the neuron gene signature. Curiously, these changes were more evident in the hippocampus and substantia nigra, regions where AD and PD pathology seem to begin. Therefore, they have highlighted the likely involvement of human astrocyte heterogeneity in predicting the regional selective vulnerability in aging and age-related diseases, such as AD and PD.

Altogether, the fact that astrocyte reactivity and dysfunction play a key role in AD and PD pathology make these cells interesting targets for the actions of synthetic and natural compounds in ameliorating cognitive performance or even restoring brain function in disease contexts, based on astrocyte biology.

## Author Contributions

All authors wrote sections of the manuscript and contributed to manuscript revision. All authors approved the submitted version of the manuscript.

## Conflict of Interest Statement

The authors declare that the research was conducted in the absence of any commercial or financial relationships that could be construed as a potential conflict of interest.

## Abbreviations

AD: Alzheimer’s disease
Aldh1L1: aldehyde dehydrogenase 1 family, member L1
ALS: amyotrophic lateral sclerosis
ATP: adenosine triphosphate
AQP4: aquaporin-4
APs: amyloid plaques
Aβ: amyloid-β
AβO: Aβ oligomers
BBB: blood-brain barrier
BDNF: brain-derived neurotrophic factor
BLBP: brain lipid binding protein
CaM-kinase: Ca^2+^/calmodulin-dependent protein kinase
CNS: central nervous system
Cx30: connexin-30
Cx43: connexin-43
C1q: complement component 1q
C3: complement component 3
C4b: complement component 4b
EAAT1: excitatory amino acid transporter 1
EAAT2: excitatory amino acid transporter 2
ER: endoplasmic reticulum
GABA: gamma-aminobutyric acid
GFAP: glial fibrillary acidic protein
GLAST: astrocyte-specific glutamate-aspartate transporter
GLP1R: glucagon-like peptide-1 receptor
GLT-1: glutamate transporter-1
GDNF: glial-derived neurotrophic factor
GS: glutamine synthetase
HD: Huntington’s disease
IFs: intermediate filaments
IFN-γ: interferon-γ
IL-6: interleukin-6
IL-8: interleukin-8
IL1R1: interleukin 1-receptor 1
IL-1α: interleukin-1α
IL-1ß: interleukin-1ß
JAK2: janus kinase 2
K_ir_4.1: inwardly rectifying potassium channel 4.1
LPS: lipopolysaccharide
LTD: long-term depression
LTP: long-term potentiation
NGF: nerve growth factor
PD: Parkinson’s disease
RG: radial glia cells
SNpc: substantia nigra pars compacta
SPARC: secreted protein-acidic and rich in cysteine
STAT3: signal transducer and activator of transcription 3
TGF-β: transforming growth factor-β
TGF-β1: transforming growth factor-β1
TLR4: toll-like receptor 4
TNF-α: tumor necrosis factor-α
TNT: tunneling nanotubes
TSP: thrombospondin
TSP-1: thrombospondin-1
WHO: World Health Organization.
